# Epigenetics, ovarian cell plasticity, and platelet-rich plasma: Mechanistic theories

**DOI:** 10.1530/RAF-22-0078

**Published:** 2022-10-18

**Authors:** E Scott Sills, Samuel H Wood

**Affiliations:** 1 Office for Reproductive Research, Center for Advanced Genetics/FertiGen, San Clemente, California, USA; 2 Regenerative Biology Group, Fertility Reserve Bank San Clemente, California, USA; 3 Gen 5 Fertility Center, San Diego, California, USA

**Keywords:** menopause, PRP, ovarian reserve, epigenetics

## Abstract

Ovarian platelet-rich plasma (PRP) is claimed to restore the fertility potential by improving reserve, an effect perhaps mediated epigenetically by platelet-discharged regulatory elements rather than gonadotropin-activated G-protein coupled receptors, as with stimulated *in vitro* fertilization (IVF). The finding that fresh activated platelet releasate includes factors able to promote developmental signaling networks necessary to enable cell pluripotency tends to support this theory. The mechanistic uncertainty of intraovarian PRP notwithstanding, at least two other major challenges confront this controversial intervention. The first challenge is to clarify how perimenopausal ovarian function is reset to levels consistent with ovulation. Perhaps a less obvious secondary problem is to confine this renewal such that any induced recalibration of cellular plasticity is kept within acceptable physiologic bounds. Thus, any ‘drive’ to ovarian rejuvenation must incorporate both accelerator and brake. Ovarian aging may be best viewed as a safeguard against pathologic overgrowth, where senescence operates as an evolved tumor-suppression response. While most ovary cells reach the close of their metabolic life span with low risk for hypertrophy, enhanced lysosomal activity and the proinflammatory ‘senescence-associated secretory phenotype’ usually offsets this advantage over time. But is recovery of ovarian fitness possible, even if only briefly prior to IVF? Alterations in gap junctions, bio-conductive features, and modulation of gene regulatory networks after PRP use in other tissues are discussed here alongside early data reported from reproductive medicine.

## Lay summary

Platelet-rich plasma (PRP) is a naturally occurring blood product which has been used to accelerate would healing and correct local aging processes. Recently, PRP has been suggested as an experimental treatment for ‘ovarian rejuvenation’ by injection into older or non-responsive ovaries. This matters because, for now, hormone replacement and in vitro fertilization/egg donation are the only available clinical treatment options for menopause and infertility. Although the gradual loss of ovarian function with increasing age is expected, it might be modified by intraovarian PRP. Indeed, return of menstruation, ovulation, and healthy term deliveries reported after ovarian PRP have moved this treatment beyond the proof-of-concept stage. But how does it actually work, and how might it be validated? Although prior study has focused on platelet cytokine signaling and ovarian stem cells, this proposal discusses new theories for alternative pathways likely to reset the ovarian biological clock.

## Introduction

Among the growing list of inputs necessary for folliculogenesis, transforming growth factor-beta 1, growth differentiation factor-9 (GDF-9), vascular endothelial growth factor (VEGF), and insulin-like growth factor-1 (IGF-1) are probably the best known. Platelet-derived growth factor and bone morphogenic proteins also appear central to cell migration, vascular support, and general ovarian function (Sills & Wood 2019). It has been shown that when specified cell exomes releasing these substances are administered near undifferentiated oocyte stem precursors, in an experimental murine ovarian insufficiency model follicle development and restoration of reproductive capacity occur (Li *et al.* 2021). Likewise, direct injection of activated platelet-rich plasma (PRP) or its derived, condensed plasma cytokines into older ovarian tissue of infertility patients may recover menses, evoke spontaneous ovulation, and enable term live birth (Sills *et al.* 2018, 2019). When PRP is activated by calcium, this causes discharge of highly purified unbound growth factors along with exosomes. In contrast, insertion of resting (non-activated) platelets for intraovarian use may reduce or even negate the post-treatment response (Rickers & Sills 2022). Since platelet exosome traffic typically accounts for <0.3% of the total protein released in activated PRP (Saumell-Esnaola *et al.* 2022), this probably adds little to the overall clinical response. This effect (H) of Ca^2+^ on efflux has been estimated to describe vesicular flow and cytokine moieties leaving activated platelets, as follows:


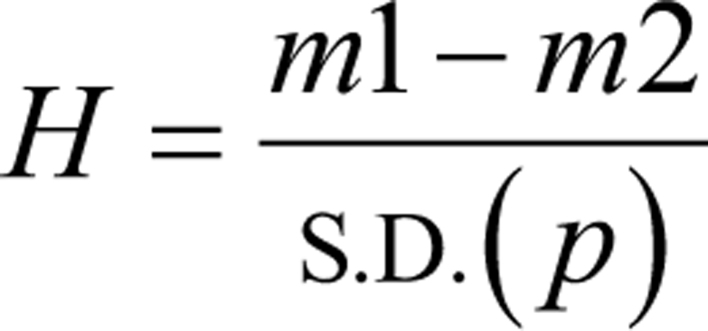



where *m*1−*m*2 is the difference in mean, and s.d.(*p*) is the pooled/weighted s.d. (Hedges 1981, Saumell-Esnaola *et al.* 2022). Modeling the microparticle dynamic entangled with activation is useful despite the biochemical composition of platelet releasate being, for now, only partially known. The current work places the processes, receptors, pathways, or combinations of these routes into a theoretical framework consistent with downstream biological observations after autologous fresh intraovarian PRP use.

## PRP – *ovaria in aeternum*?

In an experimental mammal bone marrow-derived mesenchymal stem cell model, investigators from Jiangsu University and Harvard Medical School (Zhang *et al.* 2020) found that PRP exposure caused the upregulation of key pluripotency genes such as *Oct4, Sox2, Sall4,* and *Nanog*. These derivatives show overlap with the set of four genes (*c-Myc, Oct3/4, Klf4,* and *Sox2*) which can put somatic cells back on a pluripotent lineage (Takahashi & Yamanaka 2006). Notably, researchers also reported reduced post-PRP expression of β-galactosidase – a known senescence biomarker after PRP use (Zhang *et al.* 2020).

The reason* Oct4* is well placed as a leading contributor to ovarian remodeling is that human gonadal (testicular) cells express *Oct4* (and *c-KIT*) after *in vitro* culture with PRP (Khadivi *et al.* 2020). It is plausible that a parallel response occurs in adult ovarian tissues following intraovarian activated platelet-derived cytokines (Sills & Wood 2022). Mao *et al.* (2014) tested the hypothesis that granulosa cells might display pluripotency by a novel shortcut using fewer regulatory factors. Indeed, they succeeded in generating this at high efficiency with only two factors, Oct4 and Sox2 (Mao *et al.* 2014). The only known factors that might substitute for Oct4 in human cellular reprogramming are a synthetic fusion protein of the lineage specifier GATA3-VP16 (but with much lower yield than Oct4, mir302/367, or mir200c/302/369) (Miyoshi *et al.* 2011, Montserrat *et al.* 2013).

The lack of evidence for a naturally occurring transcription factor capable of promoting *Oct4* suggests that there are some uncharacterized factors which are essential for proper reprogramming (Mai *et al.* 2018), a conclusion which encourages research focusing on numerous still-incompletely defined PRP components.

## PRP and DNA methylation

Epigenetic processes modulate gene expression without changing the basic DNA sequence; specific genes are silenced via transcriptional repression during differentiation and growth (Smith & Meissner 2013). This is done by adding or removing chromatin modifications by posttranslational modification (Bannister & Kouzarides 2011). Examples include acetylation, methylation, or phosphorylation which encounter protein complexes to direct gene expression (Li & Li 2021). In mammals, this steric adjustment usually involves methylation at cytosine or adenine nucleobases. For cytosine, its conversion to 5-methylcytosine (5mC) usually yields repression, while adenine becoming N6-methyladenine is associated with gene amplification (Yasmin *et al.* 2015, Deniz *et al.* 2019). Epigenetic reprogramming in mammals is accomplished in two phases to reset the pluripotent potential by clearing away prior patterns governing cell lineage. One of these steps occurs somatically, while the second operates in the germline. In this way, all previous DNA methylation is erased to establish a new, *de novo* pattern for restoration of the epigenome (Sendžikaitė & Kelsey 2019, Greenberg & Bourc'his 2019).

If pregnancy were established by a young mother, such offspring would derive from an oocyte typified by a partially open chromatin structure (Schächner *et al.* 2022). However, if conception were delayed much beyond 35 years of age that oocyte will be substantially different, characterized by sharply reduced 5mC in the egg genome. This constrains repression of transposable elements (Yoder *et al.* 1997), placing insertional mutations within coding and/or regulatory sequences to accumulate as net genomic instability (López-Otín *et al.* 2013). Indeed, the mature human oocyte released at 30 years of age already has at least 1.5× more transposable elements than at 20 years of age (Sturm & Vellai 2022). This mutagenesis tracks to senescence and, over time, leads to loss of the affected cell (Sturm *et al.* 2017, Gorbunova *et al.* 2021).

An intriguing feature common among apparently ageless and potentially immortal eukaryotic systems (i.e. cnidaria, planarian flatworm) is ‘P‐element‐induced wimpy testis in Drosophila’ – Piwi‐interacting noncoding RNA (Piwi-piRNA) pathway (Sturm *et al.* 2017). This gene regulator squelches mobile transposable elements which move from one locus to another, resulting in mutation. These 'jumping genes' amplify with old age, underscoring their role in genetic information decay with advanced age (Sturm *et al.* 2017). In contrast, planarian flatworms and freshwater hydra enjoy seemingly unlimited self-renewal by embodying components of this Piwi‐piRNA mechanism (Petralia *et al.* 2014).

In adult human ovarian tissue, characterization of Piwi‐piRNA is preliminary, although basic aspects have already been discovered in testes (Moody *et al.* 2020). Present in hematopoietic stem cells and certain malignancies, Piwi-piRNA engages to produce essentially unbounded proliferation and renewal. Notably, where the Piwi‐piRNA pathway is inactive, significant intron error accrues (Sturm *et al.* 2017). Once dismissed as ‘junk DNA’, introns and their alterations are associated with normal aging and associated pathologies (Huang *et al.* 2012, Kreiling *et al.* 2017). If retrotransposon dynamics rise exponentially with age, the uptick in copy number would align with the mortality pattern observed in many animal species (Sturm *et al.* 2017).

**Figure 1:**
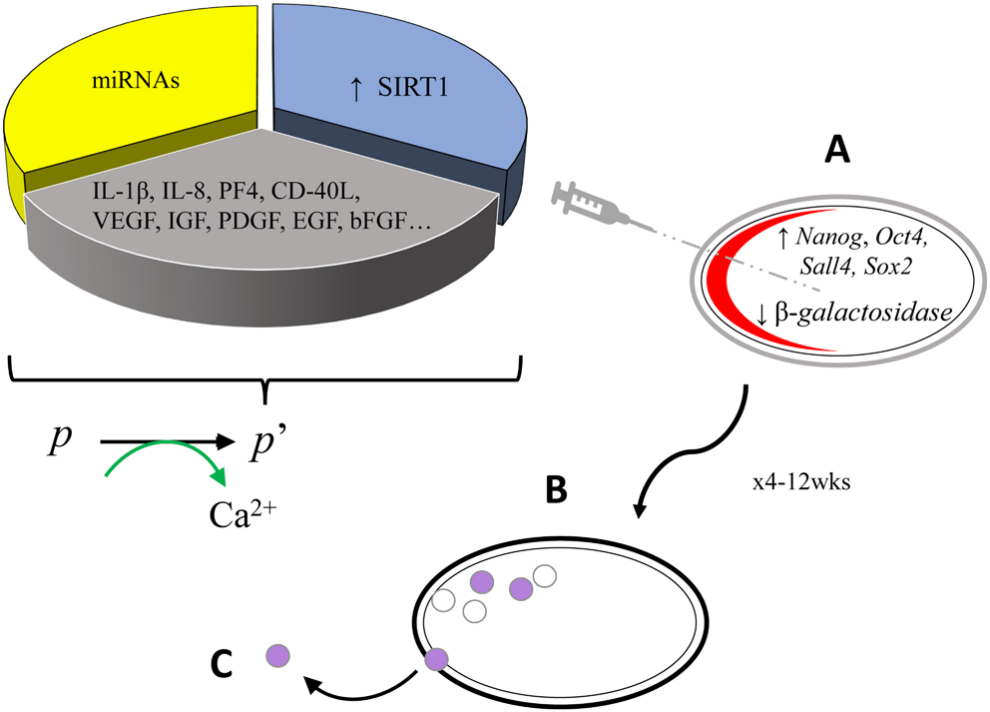
Fresh autologous platelet-rich plasma (*p*) is activated (green) to produce *p*’, enabling platelet releaseate with at least three constituents – miRNAs, multiple cytokines/growth factors, as well as gene regulators targeting undifferentiated oocyte precursors. Via single-needle puncture, *p*’ is injected into ovarian stroma (A) and subcapsular segments (red). Serum AMH is periodically monitored [3] using a uniform assay for documentation of ovarian reserve response (B), leading to follicular development (purple) and occasional spontaneous ovulation (C).

Genome-wide analysis of rat DNA methylation patterns for genes relevant to hormone control, glutamate signaling, and melatonin and circadian pathways suggests that menopause onset is under epigenetic control (Bacon *et al.* 2019). Moreover, histone and methylation changes occur across the perimenopause transition, suggesting epigenetic reorganization begins well ahead of menopause typified by loss of plasticity potential (Yin *et al.* 2015, Bacon *et al.* 2019). Activity of DNA methyltransferases is at least partially under the control of miRNAs, supplied as a constituent of activated PRP (see Fig. 1). These neuroendocrine studies encourage ongoing parallel investigations in a human ovary setting.

### Measuring menopause and how to reset it

As menopause nears, it brings a maternal refractoriness to gonadotropins in metabolically depleted ovaries which renders oocyte recruitment during IVF essentially futile. The static count for resting/non-growing follicles declines sharply with age, and this loss has been estimated within the entire ovary field (Hansen *et al.* 2008). Setting mean menopause age at 51 years, less than 800 resting follicles are predicted to remain in both ovaries at menopause, with 95% CI for age of menopause between 41.9 and 57.8 years:

log (*n*) = (–0.00019) × *ay* ^2.452^ + 5.717

where *ay* = age in years. Here, no sudden exhaustion is described but rather a constantly increasing decay rate. The model not only agrees well with observed ages of menopause but shows that a considerable percentage of follicle decline cannot be explained by age alone. Even when limited to primordial follicles, the model is still a better fit to the data than any previous model (Hansen *et al.* 2008). The accompanying perimenopausal neuroendocrine milieu characterized by chronic follicle stimulating hormone (FSH) elevation would seem to undercut the clinical rationale for administering still more (high dose and synthetic) gonadotropins, unless recapture of gonadal sensitivity could somehow be produced first.

If ovarian stem-like cells are resident in the subcapsular layer even in older/menopausal ovaries (Sills & Wood 2019), these important targets would remain indifferent to conventional IVF stimulation until proper recruitment and development signals are communicated first. As reported previously, interleukin-7 (IL-7) starts a signaling cascade favoring downstream release of interferon-γ (IFN-γ), tumor necrosis factor-α (TNF-α), and IL-10, which then regulate genes for oocyte maturation (Zhang *et al.* 2020, Sills & Wood 2022). In contrast, GDF-15, IFN-γ, insulin-like growth factor-binding protein-2 (IGFBP-2), IGFBP-7, IL-15, IL-1β, IL-17A, IL-8, monocyte chemoattractant protein-1 (MCP-1), tissue inhibitors of metalloproteinase-2, TNF-α, VEGF-A, and interferon-inducible protein-10 have been associated with aging ovarian status (Shin *et al.* 2022). When the oocyte-specific homeobox gene (*Nobox*) is muted, the postnatal oocyte pool is severely curtailed in mice by interrupting the transition from primordial to growing follicles. Echoing features seen in human menopause and low ovarian reserve, murine follicles are overtaken by fibrosis if *Nobox* is absent. Indeed, Rajkovic *et al.* were among the first to show how *Nobox* knockout causes sharp downregulation of *Oct4* and *Gdf9*, genes known to be preferentially expressed in murine oocytes (Rajkovic *et al.* 2004). These pluripotency markers (with *Sox2, Sall4,* and *Nanog*) are amplified following local intraovarian PRP dosing (Zhang *et al.* 2020).

Interestingly, recent cross-species study reported on uterine lavage using human PRP to treat mouse intracavitary uterine synechiae (Kim *et al.* 2022), where significantly increased STAT3 phosphorylation (a critical transcription factor in tissue regeneration) was observed with upregulated Mt2-mmp, Lox, and Adm, all mediators of tissue remodeling. Cell proliferation, maintenance of genome stability, and metabolic regulation are all influenced by sirtuins (Nielsen *et al.* 2022), which are NAD-dependent deacetylases. Investigators at Huazhong Science & Technology University discovered that SIRT1, SIRT3, and SIRT6 are closely related to ovarian reserve and may serve as proxy markers of ovarian aging (Zhang *et al.* 2016). Researchers from Jilin University later carried this forward to show activated PRP accelerates differentiation and viability of specific cell types via SIRT1 (Xu *et al.* 2021). Recording how autologous activated PRP affects the SIRT group specifically in the adult human ovary is planned.

## Cellular conductance and bioelectric considerations

Specialized protein channels are required to facilitate fertilization, immune response, and cellular bioelectric status, as free ion migration is checked by hydrophobic lipid membranes (Gadsby *et al.* 2009). For human luteinized granulosa cells, potassium and sodium channels are homologous with those in neuroendocrine cells of adrenal and thyroid C cells (Klugbauer *et al.* 1995, Bulling *et al.* 1999). How these ion gates change within ovaries during aging is being studied, but manipulation of cell cycle regulators via redox inputs and voltage gradients has been shown to influence rejuvenation elsewhere (Durant *et al.* 2017, Weng *et al.* 2021, Bakalova *et al.* 2022).

For example, intercellular gap junction manipulations can adjust cell polarity and potentiate signal transmission capable of dramatic reprogramming of biological morphologies (Bischof *et al.* 2020, Cervera *et al.* 2021). Phenotypic consequences for senescent ovary systems have not yet been fully described but are expected to mimic previously reported regeneration to reestablish function, reset a distributed goal state, and correct large-scale anatomies and/or functions from initial conditions (Pezzulo *et al.* 2021). How is this possible? Ongoing transcriptional systems are highly plastic and retain much potential for remodeling, yet little is known about this ‘bioelectrostat’ which may be miscalibrated either too high or too low. Disordered transmembrane ion traffic is well researched in cardiac disease, schizophrenia, bipolar disorder, and Alzheimer's disease (Berridge 2012) but not yet in ovarian senescence.

Also of importance are cellular water levels, which fluctuate over time as with electrolytes. In female reproduction, aquaporins manage water transport at intraluminal, interstitial, and capillary levels, largely under progesterone direction (Kordowitzki *et al.* 2020). Previous work has shown that at least 11 aquaporin isoforms operate in the female reproductive tract of various mammals, including human. The first aquaporin in the female reproductive system was confirmed by isolating cDNA encoding for a water channel generated from (human) uterus where cloned cDNA showed close homology (99.8%) with the 28 kDa human erythrocyte CHIP28 (Agre *et al.* 1993). While the function of transmembrane channels is subject to resting voltage potentials (Bulling *et al.* 1999), how platelet (PLT) cytokines/PRP may impact ovarian cell membrane portal dynamics is only now being investigated. This should extend the fibroblast work ofSassoli *et al.* (2022) who were the first to observe a mechanism involving gap junction currents as operational targets for selected platelet cytokines.

## Conclusions

One reason for age-associated ovarian disability could be due to gradual maladaptive epigenetic noise which gathers over time to dysregulate gene expression, culminating in the familiar ‘poor ovarian reserve’ picture commonly seen in clinical IVF practice. As human ovarian function declines with age, resetting plasticity via cell development switches actuated by external inputs might be within reach – as we propose here. This is less revolutionary than at first it may seem. The chronicle of biological science sometimes skips the chapter on phage λ lytic cycle regulation, now an obscure example of plasticity mapping (Ptashne 1992, Sommer 2020). Documenting how autologous PRP could supply the essential stimulus to reset ovarian plasticity is the topic of current studies.

While the subject of how ovary cells cease their growth once an optimal point has been reached frames a central tenet of developmental biology, it remains incompletely understood. For plants, root growth cessation requires action of an auxin influx carrier, AUX1 (Liu *et al.* 2022). For animals, miRNAs regulate various biological processes including apoptosis and proliferation (Gareev *et al.* 2021). Because at least 60% of human protein-coding genes are directly regulated by miRNAs (Dexheimer & Cochella 2020) and most circulating miRNAs are of platelet origin (Czajka *et al.* 2021), the behavior of this PRP component within human ovary is an attractive topic for research (Sills & Wood 2022).

Understandably, there is hope that such searches for alternatives to oocyte donation, IVF, synthetic hormones, or gonadotropins will gain validation. Resumed menses after menopause or deliveries for infertility patients after intraovarian PRP have drawn scrutiny on this experimental procedure. While case reports and small series are encouraging, strong evidence to support its general application is missing (Kheil *et al.* 2022). But to claim the topic is unimportant to women’s reproductive health seems incorrect, as a recent survey of world output on intraovarian PRP research (Sills & Wood 2021) was soon eclipsed by new datasets published from leading centers. For example, Australian experts reported on 20 consecutive IVF patients with severely reduced reserve to compare outcomes before vs after intraovarian PRP (Tremellen & Pacella-Ince 2022). Following non-activated PRP administered via 19G needle performed under sedation, IVF was completed 2––3 months later. Among six patients achieving pregnancy, the mean age was >40 years; there were no losses by 20 weeks gestation and two have since delivered (Tremellen & Pacella-Ince 2022). A protocol from New York included 20G needle injection of non-activated PRP with up to 12 punctures per ovary, followed by stimulated IVF cycles after ~1 month (Barad *et al.* 2022). Close endocrine monitoring of 80 cases revealed no clinically significant effects on ovarian function, but two deliveries occurred (both patients’ age = 40 years) during an impressive 1-year follow-up interval (Barad *et al.* 2022). Perhaps the largest single-center intraovarian PRP experience to date (*n* = 510) originates from Istanbul (Cakiroglu *et al.* 2022) where autologous PRP (activation unspecified) was injected via 17G needle under sedation. The mean patient age in the series was 40.3 years, with post-PRP pregnancy and live birth rates of 20.5 and 12.9%, respectively (Cakiroglu *et al.* 2022).

These outcomes – regardless of which IVF calendar or what needle is used for PRP injection – suggest that such details may be subsidiary so long as clinics follow protocols most familiar to them (Sills *et al.* 2018). Descriptive reports can add to the PRP knowledge fund, as interested researchers gain a better understanding of methods and techniques for ‘ovarian rejuvenation’.

## Declaration of interest

ESS has been awarded U.S. Trademark #88505430 for treatment of female hormone and fertility enhancement that utilizes a specified method of intraovarian injection of autologous PRP.

## Funding

None.

## Author contribution statement

ESS developed the research plans and initial drafts; ESS and SHW reviewed the literature and edited the work. Both authors read and approved the final manuscript.
